# Optimized grouping to increase accuracy of prediction of breeding values based on group records in genomic selection breeding programs

**DOI:** 10.1186/s12711-019-0509-z

**Published:** 2019-11-15

**Authors:** Thinh T. Chu, John W. M. Bastiaansen, Peer Berg, Hans Komen

**Affiliations:** 1 Center for Quantitative Genetics and Genomics, Aarhus University, 8830 Tjele, Denmark; 2 Animal Breeding and Genomics, Wageningen University & Research, 6709 PG Wageningen, The Netherlands; 3 Faculty of Animal Science, Vietnam National University of Agriculture, Gia Lam, Hanoi, Vietnam; 4 Department of Animal and Aquacultural Sciences, Norwegian University of Life Sciences, 1432 Aas, Norway

## Abstract

**Background:**

Phenotypic records of group means or group sums are a good alternative to individual records for some difficult to measure, but economically important traits such as feed efficiency or egg production. Accuracy of predicted breeding values based on group records increases with increasing relationships between group members. The classical way to form groups with more closely-related animals is based on pedigree information. When genotyping information is available before phenotyping, its use to form groups may further increase the accuracy of prediction from group records. This study analyzed two grouping methods based on genomic information: (1) unsupervised clustering implemented in the STRUCTURE software and (2) supervised clustering that models genomic relationships.

**Results:**

Using genomic best linear unbiased prediction (GBLUP) models, estimates of the genetic variance based on group records were consistent with those based on individual records. When genomic information was available to constitute the groups, genomic relationship coefficients between group members were higher than when random grouping of paternal half-sibs and of full-sibs was applied. Grouping methods that are based on genomic information resulted in higher accuracy of genomic estimated breeding values (GEBV) prediction compared to random grouping. The increase was ~ 1.5% for full-sibs and ~ 11.5% for paternal half-sibs. In addition, grouping methods that are based on genomic information led to lower coancestry coefficients between the top animals ranked by GEBV. Of the two proposed methods, supervised clustering was superior in terms of accuracy, computation requirements and applicability. By adding surplus genotyped offspring (more genotyped offspring than required to fill the groups), the advantage of supervised clustering increased by up to 4.5% compared to random grouping of full-sibs, and by 14.7% compared to random grouping of paternal half-sibs. This advantage also increased with increasing family sizes or decreasing genome sizes.

**Conclusions:**

The use of genotyping information for grouping animals increases the accuracy of selection when phenotypic group records are used in genomic selection breeding programs.

## Background

Continuous individual records are difficult and expensive to obtain for some economically important traits such as feed efficiency and egg production. For breeding programs that aim at improving genetic gain in the presence of genotype-by-environment (G × E) interactions, continuous individual recording of such traits can even be impossible for animals that are tested in commercial production environments or in village conditions. In such situations, the use of pooled data from group records can be an alternative, which was shown to be feasible for predicting variance components and breeding values of animals with pedigree-based best linear unbiased prediction (BLUP) [1–4]. These studies showed that accuracy of prediction depends on the relationships between group members and increases when group members are more closely related. In the additive numerator relationship matrix, the relationship coefficients between members of a full-sib group are all the same, i.e. 0.5 for unrelated parents or higher for inbred parents. Compared to pedigree information, genomic information gives a better measure of the relationships between animals. VanRaden [5] showed that the realized genomic relationships between specific pairs of full-sibs vary with the standard deviation of the relationship coefficients according to genome size and number of chromosomes. Empirical values of the relationship coefficients between pairs of full-sibs range from 0.27 to 0.70 for chicken [6] and from 0.35 to 0.65 for cattle [7]. We hypothesized that genomic information could be exploited to improve the accuracy of prediction of breeding values based on group records when it is available before animals are grouped for phenotype testing.

The accuracy of prediction of breeding values based on group records increases as the relationships between animals within groups increase [1–3], thus an increase in genomic similarity between group members may improve accuracy of prediction. Unsupervised clustering of genetically similar individuals into groups based on genomic data is implemented in a program named STRUCTURE [8]. This Bayesian, model-based program, is widely used in analyses of population structure [8]. This program integrates over the parameter space, infers population structure, and makes cluster assignments for every individual [8]. The number of subpopulations, or clusters, can be given or estimated. An output of the program is the membership coefficient or probability that an individual belongs to a given cluster. Clustering individuals into subpopulations is a similar concept to clustering animals that have close relationships into the same groups. However, this approach may not be optimal for designing breeding programs because the number of individuals assigned to each cluster can vary under unsupervised clustering. For example, the breeding facilities, which are usually fixed systems, may be able to accommodate four groups with four animals per group, but unsupervised clustering ends up with animals that are clustered into only three groups of different sizes. Besides, the membership coefficient of an animal based on STRUCTURE cannot always clearly distinguish to which group the animal belongs.

Thus, in addition to the grouping method based on the STRUCTURE program, we propose a grouping method that maximizes the relationships between animals within a group, based on the realized genomic relationship matrix. This grouping method is a supervised clustering approach, in which the number of groups and group sizes are defined as fixed input variables. Instead of using genotyping data directly, the method uses it indirectly through the realized genomic relationship matrix.

The objectives of our study were to: (1) compare these two grouping methods based on genotyping information to improve accuracy of selection with group records, when using genomic BLUP (GBLUP) models to estimate variance components and genomic breeding values (GEBV) with group records, and (2) investigate the effects of adding surplus genotyped offspring, of the number of groups, family sizes and genome sizes on breeding schemes that used the proposed grouping method. These grouping methods were tested with a stochastic simulation study.

## Methods

We simulated a population of animals in three steps: (1) simulation of individual genotypes and phenotypes using the stochastic simulation program ADAM [9], (2) allocation of simulated animals into groups based on pedigree or genomic information using different grouping methods, and (3) calculating group phenotypes from individual phenotypes. Variance components and breeding values were estimated from individual records or from group records using a GBLUP model.

### Breeding schemes, genotype and trait simulations

The historical base population and its genomic structure were from Chu et al. [10] with a simulated genome of 26 chromosomes and a total length of 916 cM. Segregating loci of 2 k quantitative trait loci (QTL) and 40 k neutral markers that were randomly distributed along the genome were used to simulate information on traits and genotypes, respectively. The segregating QTL and markers had a minor allele frequency of at least 0.05 in the base population. Inheritance of QTL and markers from parents to descendants followed the standard principles of Mendelian inheritance, and allowed for recombination as described in Chu et al. [10]. From the base population, 20 sires and 200 dams were used for a nested mating scheme in which one sire is mated with 10 dams. Each dam produced 16 offspring, thus the total number of offspring was 3200. Sex was randomly assigned to the offspring with a 1:1 ratio. Only one generation of offspring was simulated. All sires, dams and offspring had genotype information.

The true breeding value of each individual was the sum of the effects of the QTL. Allele substitution effects of QTL were randomly sampled from a normal distribution ${\text{N}}\left( {0, 1} \right)$, and then rescaled to achieve the initial additive genetic variance of 0.3 in the base population. The simulated phenotype of individual records was the sum of the true breeding value and an environmental deviation term:

$$y_{i} = \mu + tbv_{i} + e_{i}$$

where $y_{i}$ is the individual phenotypic record of animal $i$; $\mu$ is the mean of the trait equal to 0; $tbv_{i}$ and $e_{i}$ are the true breeding value and residual environmental deviation term of animal $i$, respectively. The residual environmental deviations were drawn from the normal distribution ${\text{N}}\left( {0, 0.7} \right)$. Thus, the phenotypic variance was 1.

Four animals were pooled per group, which resulted in 800 groups. A group record was composed of the sum of all records of the group members.

### Estimation of variance components and prediction of breeding values

Individual records and group records were used to estimate variance components and GEBV using the DMUAI module from the DMU software package [11]. The model for individual records in matrix notation was (GBLUP_i_: Eq. 1):

1 $${\mathbf{y}} = {\mathbf{1}}\mu + {\mathbf{Zg}} + {\mathbf{e}}\text{,}$$

where ${\mathbf{y}}$ is a vector of phenotypic individual records; $\mu$ is the mean; ${\mathbf{g}}$ and ${\mathbf{e}}$ are vectors of additive genetic and residual effects, respectively. These vectors are assumed to be normally distributed: ${\mathbf{g}}\sim {\text{N}}\left( {0, {\mathbf{G}}\sigma_{{\mathbf{g}}}^{2} } \right)$ and ${\mathbf{e}}\sim {\text{N}}\left( {0, {\mathbf{I}}\sigma_{{\mathbf{e}}}^{2} } \right)$, where ${\mathbf{G}}$ is a genomic relationship matrix constructed from marker data and **I** is an identity matrix associating residuals to individual phenotypic records; $\sigma_{{\mathbf{g}}}^{2}$ and $\sigma_{{\mathbf{e}}}^{2}$ are the additive genetic variance and residual variance, respectively. The incidence matrix ${\mathbf{Z}}$ links ${\mathbf{g}}$ to individual phenotypic records.

When group records were analyzed, the model for estimating variance components and GEBV was similar to the exact model of Olson et al. [3] and to the model of Su et al. [2], except that a realized genomic relationship matrix was used instead of the additive numerator relationship matrix. The models applied to equal group sizes was (GBLUP_gr_: Eq. 2):

2 $${\mathbf{y}}^{*} = {\mathbf{1}}\mu + {\mathbf{Z}}^{*} {\mathbf{g}} + {\mathbf{e}}^{*} ,$$

where ${\mathbf{y}}^{*}$ is a vector of group records, i.e. the sum of the individual’s phenotypes, with number of elements equal to number of groups; ${\mathbf{g}}$ is a vector of additive genetic values of individuals as described above in the model for individual records: ${\mathbf{g}}\sim {\text{N}}\left( {0, {\mathbf{G}}\sigma_{{\mathbf{g}}}^{2} } \right)$; ${\mathbf{e}}^{*}$ is a vector of residuals: ${\mathbf{e}}^{*} \sim {\text{N}}\left( {0, {\mathbf{R}}\sigma_{{\mathbf{e}}}^{2} } \right)$, where ${\mathbf{R}}$ is a diagonal matrix and diagonal elements are equal to group size. Matrix ${\mathbf{Z}}^{*}$ is an incidence matrix associating ${\mathbf{g}}$ to phenotypic group records. Matrices ${\mathbf{Z}}$ and ${\mathbf{Z}}^{*}$ have an equal number of columns but a number of rows equal to the number of individual records and group records, respectively.

For example, eight animals numbered 3 to 10 that were offspring of animals 1 and 2 were grouped in two groups of four animals. Phenotypic records of groups were 2.6 and 3.5. The model for group records is:

$$\left[ {\begin{array}{*{20}c} {2.6} \\ {3.5} \\ \end{array} } \right] = \left[ {\begin{array}{*{20}c} 1 \\ 1 \\ \end{array} } \right]\mu + \left[ {\begin{array}{*{20}c} 0 & 0 & 1 & 1 & 1 & 1 & 0 & 0 & 0 & 0 \\ 0 & 0 & 0 & 0 & 0 & 0 & 1 & 1 & 1 & 1 \\ \end{array} } \right]\left[ {\begin{array}{*{20}c} {g_{1} } \\ {g_{2} } \\ {g_{3} } \\ \ldots \\ {g_{10} } \\ \end{array} } \right] + \left[ {\begin{array}{*{20}c} {\mathop \sum \limits_{i = 3}^{6} e_{i} } \\ {\mathop \sum \limits_{i = 7}^{10} e_{i} } \\ \end{array} } \right] .$$

The realized genomic relationship matrix ${\mathbf{G}}$ was constructed from marker data of all sires, dams and offspring individuals using VanRaden’s [12] method 1:

$${\mathbf{G}} = \frac{{{\mathbf{MM^{\prime}}}}}{{2\sum p_{j} \left( {1 - p_{j} } \right)}},$$

where ${\mathbf{M}}$ is a matrix that has number of rows equal to number of animals and number of columns equal to number of markers; matrix ${\mathbf{M}}$ is centered so that elements in column $j$ are 0-2$p_{j}$, 1-2$p_{j}$ and 2-2$p_{j}$ for genotypes $A_{1} A_{1}$, $A_{1} A_{2}$ and $A_{2} A_{2}$, respectively; $p_{j}$ is the allele frequency of $A_{2}$ at locus $j$ computed from the marker data of all dams, sires and offspring. Division by $2\sum p_{j} \left( {1 - p_{j} } \right)$ scales matrix ${\mathbf{G}}$ to be analogous to the pedigree-based numerator relationship matrix. The realized genomic relationship matrix ${\mathbf{G}}$ was used in the genetic evaluation models and grouping methods and to investigate the distributions of relationships.

### Grouping methods

For group records, animals were pooled into groups based on either pedigree or genomic information. Grouping methods based on pedigree information were random grouping of full-sibs and random grouping of paternal half-sibs. The two grouping methods based on genomic information were unsupervised clustering based on genotypes and supervised clustering based on the genomic relationships.

#### Random grouping of paternal half-sibs

For random grouping of paternal half-sibs, allocation of animals into groups was based on having a common sire. From each parental half-sib group of 160 animals, individuals were randomly allocated to 40 groups.

#### Random grouping of full-sibs

For random grouping of full-sibs, allocation of animals into groups was based on having a common sire and dam. From each full-sib group of 16 animals, individuals were randomly allocated to 4 groups.

#### Unsupervised clustering based on genotypes

When unsupervised clustering analysis using the STRUCTURE program was applied to all 3200 animals, paternal half-sibs from a sire were always clustered into one group even if the assumed number of clusters was set at 800. When unsupervised clustering was applied to a group of paternal half-sibs from a single sire, full-sibs from a family were always clustered into one group. Therefore, clustering analysis was carried out separately for every full-sib family of 16 animals. The admixture model in STRUCTURE was used [8]. The number of clusters was set to 4, and allele frequencies were assumed to be correlated between clusters [13]. Cluster membership coefficients of the 16 animals from the output were used for allocation to groups. Animals from the same full-sib group were pre-allocated into four groups based on their highest membership coefficients. In many cases, the pre-allocated four groups did not all have the expected number of four animals. Four animals with the top ranking membership coefficients from the biggest group were allocated to the first group. Then, the remaining 12 animals were pre-allocated into three groups based on their highest membership coefficients. Four animals with the top ranking membership coefficients from the biggest group were allocated to the second group. Similarly, four animals were allocated into the third group, and the fourth group consisted of the remaining four animals. These grouping allocation procedures were applied to all 200 full-sib families to constitute 800 groups. With the unsupervised clustering method based on genotypes, animals within a group were always full-sibs.

#### Supervised clustering based on genomic relationships

A supervised clustering method scripted in R [14] was developed to pool four animals into groups based on realized genomic relationships between animals. Applying this approach to all 3200 offspring was time-consuming because of the many possibilities for allocating the offspring into 800 groups. The probability that half-sibs or non-related animals were placed in the same group was extremely low. Therefore, grouping was carried out separately for every full-sib family of 16 animals. In each round of iteration, animals were assigned into groups by an evolutionary algorithm as follows:

- Animals from a full-sib family were randomly assigned to four groups with four animals in each group;
- An exchange of two randomly chosen animals between two randomly chosen groups was proposed;
- Group membership was updated if the proposal resulted in an increased mean genomic relationship between members within groups;
- The round ended when the exchange of two animals between two groups did not increase the mean of genomic relationships for $n_{a}^{2} \times \sum\nolimits_{i = 1}^{{n_{g} - 1}} i$ times, where $n_{a}$ is the number of animals per group (group size) and $n_{g}$ is the number of groups per full-sib family. The exchange of two animals between two groups was a random process, but if the exchange did not increase genomic relationships between group members, these two animals were not chosen for the exchange until a new set of animal groups was formed (the solution on the set of groups was retained if there was no improvement). The $n_{a}^{2} \times \sum\nolimits_{i = 1}^{{n_{g} - 1}} i$ times were the number of possibilities of forming a new set of animal groups when two animals are randomly chosen from two random groups.

The exchange of two animals in each round of the above iteration is a conditional event given that a certain set of groups of animals is formed. Therefore, the above evolutionary algorithm was iterated for 300 rounds that formed up to 300 different sets of groups. The set of animal groups that resulted in the highest genomic relationship coefficient between group members was chosen. The number 300 is an empirical number that was chosen after different trials to get a set of animal groups with the highest genomic relationship coefficient between group members.

Through this supervised clustering method based on genomic relationships, the offspring were allocated into 800 different groups and, within each of these groups, animals were always full-sibs.

### Sensitivity analysis

To investigate the effects of adding surplus genotyped offspring (more genotyped offspring than required in the groups), number of groups for the same total number of individuals, family sizes and genome sizes for all grouping strategies (except the unsupervised clustering method), four additional simulations (SS1 to SS4) were carried out with 100 replicates for each scenario. In sensitivity analysis simulation 1 (SS1) group sizes and number of groups per full-sib family were the same as in the base scenario. The number of surplus genotyped offspring were varied, i.e. set at 16 or 32 offspring per full-sib family (Table 1). For comparisons, individual records in SS1 were obtained from only 16 offspring per dam that were randomly chosen from each full-sib family. SS2 had the same family structure as in the base scenario, but group sizes and number of groups per full-sib family were varied. In SS3, the number of groups was constant, but family size (number of offspring per dam) was varied. SS4 was the same as in the base scenario, except that the simulated genome consisted of 30 chromosomes each 100 cM long, i.e. it had a total length of 3000 cM. Table 1 summarizes the features of the base scenario and of SS1 to SS4 for the factors investigated.

**Table 1:** Surplus offspring, group sizes, number of groups and family sizes for sensitivity simulations (SS) 1 to 4

Investigated factors	Base scenario	SS1	SS2	SS3	SS4
Surplus genotyped offspring without phenotypes per full-sib family	0	16 or 32	0	0	0
Group sizes (animals per group)	4	4	2 or 8	2, 8 or 12	4
Number of groups per full-sib family	4	4	8 or 2	4	4
Family sizes (offspring per dam)	16	32 or 48	16	8, 32 or 48	16
Genome size	916 cM	916 cM	916 cM	916 cM	3000 cM

The supervised clustering method based on genomic relationships in SS1 to SS4 was similar to that in the base scenario with the aim to maximize relationships between animals within groups. In SS1, one extra group was added that included all surplus animals, which were not phenotyped or included in the calculation of the average relationship within groups. The probability of sampling groups for the exchange between two random animals corresponded to the number of animals of these groups.

### Data analysis

Scenarios were replicated 100 times. Accuracy of GEBV predictions was computed as the correlation between GEBV and true breeding values of all phenotyped offspring individuals. Bias of GEBV predictions was computed as the regression coefficient of true breeding values on GEBV. Coancestry coefficients were computed as the means of realized genomic relationships between the top GEBV rankings of 20 males and 200 females.

Pairwise genomic relationships between animals that were half-sibs, full-sibs, paternal half-sibs or genomic-close full-sibs were used to investigate the distribution of relationships. Half-sibs were offspring from the same sires, but from different dams based on pedigree. Paternal half-sibs were offspring from the same sire that could be, but not necessarily, from the same dam. Genomic-close full-sibs were full-sibs that became members of the same group after applying unsupervised clustering based on genotyping or supervised clustering based on genomic relationships. All pairwise genomic relationships of group members from all 100 replicates were combined and used to calculate means and standard deviations.

## Results

**Fig. 1:**
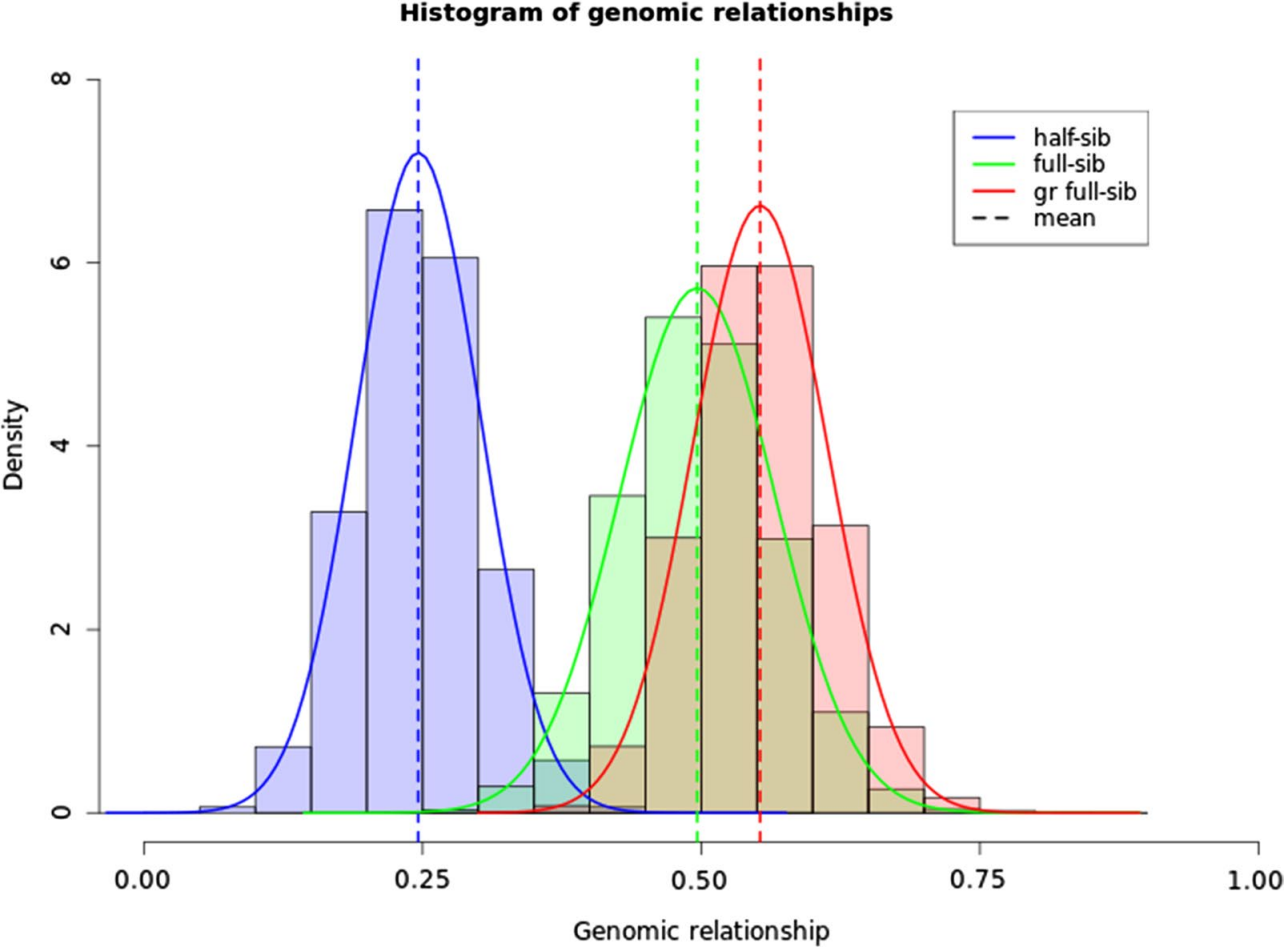
Distribution of realized genomic relationships between half-sibs (blue line and light-blue bars), full-sibs (green line and light-green bars) and genomic-close full-sibs (full-sibs grouped by the supervised clustering method based on genomic relationship (red line and pink bars). Broken vertical lines are means of the genomic relationships

Realized genomic relationships of the breeding schemes were calculated for half-sibs, paternal half-sibs, full-sibs and groups of genomic-close full-sibs that were grouped by either unsupervised clustering based on genotypes or supervised clustering based on genomic relationships (Table 2). As expected, the means of realized genomic relationships were roughly 0.50 for full-sibs and 0.25 for half-sibs (Fig. 1). Paternal half-sib relationships were a mixture of full-sib and half-sib relationships. The highest means of relationships within groups were obtained when these were grouped by supervised clustering (0.55) and by unsupervised clustering (0.54).

**Table 2:** Means and standard deviations of realized genomic relationships between half-sibs, paternal half-sibs, full-sibs and genomic-close full-sibs, which were grouped by unsupervised clustering based on genotypes or by supervised clustering based on genomic relationships

Relationships	Mean	Standard deviation
Half-sibs	0.246	0.055
Paternal half-sibs	0.270	0.093
Full-sibs	0.496	0.070
Genomic-close full-sibs grouped by supervised clustering based on genomic relationships	0.553	0.060
Genomic-close full-sibs grouped by unsupervised clustering based on genotypes	0.538	0.067

Variance components estimated from group records were consistent with those estimated from individual records (Table 3), and these estimates were not significantly different from simulated values. However, variance components estimated from group records had a higher standard deviation than those estimated from individual records.

**Table 3:** Estimates of additive genetic variance ($\sigma_{{\mathbf{a}}}^{2}$) and residual variance ($\sigma_{{\mathbf{e}}}^{2}$) (mean over 100 replicates ± standard deviation) estimated from individual records and from group records

Records	Model	$\sigma_{{\mathbf{a}}}^{2}$ (SD)	$\sigma_{{\mathbf{e}}}^{2}$ (SD)
Simulated parameters		0.30	0.70
Individual records	GBLUP_i_	0.300 (0.030)	0.698 (0.022)
Group records from supervised clustering method based on genomic relationships	GBLUP_gr_	0.302 (0.042)	0.691 (0.048)
Group records from unsupervised clustering method based on genotypes	GBLUP_gr_	0.301 (0.043)	0.693 (0.050)
Group records from random grouping of full-sibs	GBLUP_gr_	0.298 (0.045)	0.695 (0.052)
Group records from random grouping of paternal half-sibs	GBLUP_gr_	0.301 (0.062)	0.695 (0.050)

Models GBLUP_i_ and GBLUP_gr_ are GBLUP model for individual records and group records, respectively. SD is standard deviations over 100 replicates

Accuracy and bias of GEBV based on individual and group records from the different prediction models are in Table 4. As expected, the accuracy of GEBV was higher when estimated from individual records than from group records. When group records were used to predict GEBV, accuracies of GEBV depended on the grouping method applied. Accuracies of GEBV decreased as the realized genomic relationships between group members decreased. Grouping methods based on genomic information led to higher accuracies of GEBV than the random grouping methods based on pedigree information. Group records from supervised clustering based on genomic relationships led to the highest accuracy and lowest standard deviation of the accuracy compared to group records from other grouping methods. Group records from random grouping of paternal half-sibs resulted in the lowest accuracy of GEBV prediction and the highest standard deviation of the accuracy.

**Table 4:** Accuracy of GEBV, bias of prediction and coancestry coefficients of top ranking animals (mean over 100 replicates ± standard deviation) on GEBV estimated from individual records and from group records

Records	Model	Accuracy (SD)	Bias (SD)	Coancestry coefficients (SD)
Individual records	GBLUP_i_	0.825 (0.020)	1.011 (0.041)	0.036 (0.009)
Group records from supervised clustering method based on genomic relationships	GBLUP_gr_	0.762 (0.028)	1.007 (0.054)	0.041 (0.010)
Group records from unsupervised clustering method based on genotypes	GBLUP_gr_	0.758 (0.030)	1.009 (0.054)	0.041 (0.009)
Group records from random grouping of full-sibs	GBLUP_gr_	0.749 (0.032)	1.015 (0.060)	0.043 (0.010)
Group records from random grouping of paternal half-sibs	GBLUP_gr_	0.682 (0.040)	1.017 (0.092)	0.049 (0.010)

Coancestry coefficients were computed for the top 20 males and 200 females ranked by GEBV that were estimated from individual records and from group records (Table 4). Coancestry coefficients between the top animals ranked by GEBV were lower when based on individual records than based on group records and were also lower with grouping methods based on genomic information than random grouping methods based on pedigree information.

### Sensitivity analysis

In SS1 to SS4, variance components estimated from group records were consistent with those estimated from individual records. Biases of GEBV estimated from individual records and group records showed no clear difference, and regressions of true breeding values on GEBV were close to 1. However, standard deviations of variance estimates and biases of GEBV over 100 replicates were greater for group records than for individual records. The results on variance estimates for SS1 to SS4 are not shown. Genomic relationships between group members, accuracies of GEBV and coancestry coefficients of the top ranking animals were similar for scenarios in SS1 to SS4. As in the main study, the supervised clustering method based on genomic relationships generally led to higher genomic relationships between group members, higher accuracies of GEBV and lower coancestry coefficients of top ranking animals than random grouping of full-sibs and random grouping of paternal half-sibs.

In SS1, group size and number of groups per full-sib family were the same as in the base scenario, but there were surplus offspring that did not belong to any group or did not have phenotypes. With 0 (base scenario), 16 (SS1) and 32 (SS1) surplus offspring, coefficients of relationships between genomic-close full-sibs that were grouped by supervised clustering were equal to 0.55, 0.60 and 0.62, respectively (Tables 2, 5). An increased number of surplus offspring tended to increase the accuracy of GEBV that were estimated from group records of the genomic-close full-sibs (Tables 4, 5). However, a change in the number of surplus offspring had no effect on the coefficient of relationships between group members or accuracy of GEBV for scenarios with groups that were formed by random grouping of full-sibs or random grouping of paternal half-sibs. The relative increases in accuracy of GEBV from using supervised clustering based on genomic information compared to using random grouping of full-sibs were 3.9 and 4.5% when the numbers of surplus offspring were 16 and 32 per full-sib family, respectively.

**Table 5:** Genomic relationships, accuracy of GEBV, bias of prediction and coancestry coefficients for sensitivity simulation 1 when the number of surplus genotyped offspring without phenotypes per full-sib family was equal to 16 and 32

Variables	Individual records	Group records		
		Supervised clustering method	Random grouping of full-sibs	Random grouping of paternal half-sibs
Surplus offspring: 16 per full-sib family				
Genomic relationships (SD)		0.602 (0.053)	0.496 (0.070)	0.270 (0.093)
Accuracy (SD)	0.824 (0.020)	0.773 (0.028)	0.744 (0.032)	0.678 (0.040)
Bias (SD)	1.000 (0.038)	1.004 (0.055)	1.001 (0.056)	1.004 (0.098)
Coancestry coefficients (SD)	0.036 (0.008)	0.042 (0.010)	0.043 (0.009)	0.049 (0.011)
Surplus offspring: 32 per full-sib family				
Genomic relationships (SD)		0.622 (0.052)	0.497 (0.070)	0.271 (0.094)
Accuracy (SD)	0.822 (0.021)	0.776 (0.028)	0.743 (0.032)	0.677 (0.040)
Bias (SD)	1.003 (0.044)	1.006 (0.058)	1.004 (0.056)	1.008 (0.094)
Coancestry coefficients (SD)	0.035 (0.007)	0.042 (0.008)	0.043 (0.009)	0.047 (0.010)

In SS2, family size was the same as in the base scenario and kept constant, but the number of groups per full-sib family, and thus the group sizes, were varied. With group records formed by supervised clustering, random grouping of full-sibs and random grouping of paternal half-sibs, an increase in the number of groups per full-sib family led to an increase in accuracy of GEBV and a decrease in coancestry coefficients of top ranking animals (Table 6). With group records formed by supervised clustering, a change in the number of groups per full-sib family from 2 to 8 changed the coefficients of genomic relationships between group members from 0.522 to 0.589, respectively but had no effect in terms of accuracy of GEBV. The relative increases in accuracy of GEBV from using random grouping of full-sibs to using supervised clustering based on genomic information were 1.3, 1.7 and 1.4% for scenarios with 2, 4 and 8 groups per full-sib family, respectively.

**Table 6:** Genomic relationships, accuracy of GEBV, bias of prediction and coancestry coefficients for sensitivity simulation 2 with 2 and 8 groups per full-sib family

Variables	Individual records	Group records		
		Supervised clustering method	Random grouping of full-sibs	Random grouping of paternal half-sibs
Number of groups: 2 groups per full-sib family				
Genomic relationships (SD)		0.589 (0.053)	0.496 (0.070)	0.270 (0.093)
Accuracy (SD)	0.825 (0.020)	0.794 (0.025)	0.783 (0.026)	0.755 (0.030)
Bias (SD)	1.011 (0.041)	1.014 (0.046)	1.011 (0.053)	1.021 (0.065)
Coancestry coefficients (SD)	0.036 (0.009)	0.038 (0.009)	0.039 (0.009)	0.041 (0.009)
Number of groups: 8 groups per full-sib family				
Genomic relationships (SD)		0.522 (0.066)	0.496 (0.070)	0.270 (0.093)
Accuracy (SD)	0.825 (0.020)	0.736 (0.033)	0.726 (0.035)	0.620 (0.051)
Bias (SD)	1.011 (0.041)	1.017 (0.068)	1.015 (0.064)	1.038 (0.101)
Coancestry coefficients (SD)	0.036 (0.009)	0.044 (0.010)	0.045 (0.010)	0.059 (0.012)

In SS3, the number of groups was constant with four groups per family, but family sizes, and therefore group sizes, were varied. With records of groups formed by different grouping methods in SS3, an increase in the family size led to an increase in accuracy of GEBV and coancestry coefficients of the top ranking animals (Table 7). With records of groups formed by supervised clustering, an increase in the family size led to a decrease in genomic relationships between group members. The relative increases in accuracy of GEBV from using random grouping of full-sibs to using supervised clustering based on genomic information were 1.2, 1.7, 2.4 and 3.2% for scenarios with family sizes of 8, 16, 32 and 48 offspring per full-sib family, respectively.

**Table 7:** Genomic relationships, accuracy of GEBV, bias of prediction and coancestry coefficients for sensitivity simulation 3 with 8, 32 and 48 offspring per full-sib family

Variables	Individual records	Group records		
		Supervised clustering method	Random grouping of full-sibs	Random grouping of paternal half-sibs
Family size of 8				
Genomic relationships (SD)		0.565 (0.057)	0.497 (0.070)	0.268 (0.091)
Accuracy (SD)	0.770 (0.028)	0.734 (0.034)	0.726 (0.035)	0.692 (0.041)
Bias (SD)	1.007 (0.062)	1.016 (0.076)	1.012 (0.087)	1.019 (0.106)
Coancestry coefficients (SD)	0.024 (0.004)	0.026 (0.004)	0.027 (0.004)	0.028 (0.005)
Family size of 32				
Genomic relationships (SD)		0.543 (0.063)	0.497 (0.070)	0.271 (0.093)
Accuracy (SD)	0.870 (0.014)	0.785 (0.026)	0.766 (0.028)	0.674 (0.040)
Bias (SD)	1.001 (0.025)	1.003 (0.036)	1.003 (0.043)	1.000 (0.074)
Coancestry coefficients (SD)	0.049 (0.016)	0.060 (0.019)	0.063 (0.020)	0.078 (0.026)
Family size of 48				
Genomic relationships (SD)		0.539 (0.064)	0.497 (0.070)	0.271 (0.094)
Accuracy (SD)	0.893 (0.011)	0.799 (0.023)	0.774 (0.025)	0.671 (0.041)
Bias (SD)	1.000 (0.018)	1.002 (0.030)	0.993 (0.030)	0.998 (0.079)
Coancestry coefficients (SD)	0.053 (0.017)	0.068 (0.021)	0.072 (0.021)	0.091 (0.032)

Breeding structures, group sizes and family sizes of SS4 were the same as those of the base scenario, but the genome structure differed with a longer genome size, i.e. 3000 cM vs 916 cM. Accuracies of GEBV based on individual records and group records were higher in the base scenario than in SS4 (Table 8). The means of genomic relationships between full-sibs or between paternal half-sibs were similar for the base scenario and SS4, but their standard deviations were lower for SS4. Genomic relationships between genomic-close full-sibs that were grouped by supervised clustering were higher in the base scenario than in SS4. The relative increases in accuracy of GEBV from using random grouping of full-sibs to using supervised clustering based on genomic information were 1.7 and 1.3% in the base scenario and SS4, respectively.

**Table 8:** Genomic relationships, accuracy of GEBV, bias of prediction and coancestry coefficients for sensitivity simulation 4 when a genome of 30 chromosomes and 3000 cM was simulated

Variables	Individual records	Group records		
		Supervised clustering method	Random grouping of full-sibs	Random grouping of paternal half-sibs
Genomic relationships (SD)		0.532 (0.036)	0.496 (0.042)	0.269 (0.080)
Accuracy (SD)	0.729 (0.030)	0.671 (0.039)	0.662 (0.040)	0.583 (0.055)
Bias (SD)	1.016 (0.057)	0.999 (0.070)	1.001 (0.065)	1.022 (0.119)
Coancestry coefficients (SD)	0.036 (0.009)	0.042 (0.010)	0.044 (0.010)	0.054 (0.011)

## Discussion

In this work, we found that with GBLUP models, the estimated variance components were similar whether they were based on group or individual records, but their standard errors were larger for those based on group records than on individual records. These findings confirm those of previous studies that used pedigree-based BLUP models to estimate variance components based on group records [2–4]. Compared to these studies [2–4], the main modification of our model for group records was the use of a realized genomic relationship matrix instead of a numerator genetic relationship matrix. With a numerator genetic relationship matrix, full-sibs in the same group have equal EBV whereas with a genomic relationship matrix, full-sibs in the same group can have different EBV. The benefit of using genomic information over pedigree information, in terms of accuracy of prediction, has been well documented in simulation studies [15–17] and empirical studies of chicken [18–20], cattle [21, 22] and pig [17, 23, 24] breeding schemes for individual records. The increase in accuracy of GBLUP prediction obtained by using individual records is due to an improved measurement of the relationships between animals and a better prediction of the Mendelian sampling terms [16]. These advantages of using genomic information should also apply to GBLUP models for group records, and thus increase the accuracy of prediction compared to pedigree-based models.

For the same number of individuals, accuracies of GEBV based on group records were lower than those based on individual records. Coancestry coefficients of animals that were selected based on group records were also higher than those based on individual records. While the results from group records cannot compete with those from individual records, the number of phenotypes that need to be recorded differs between group and individual record data. At the commercial production environment level, group records are sometimes the only available phenotypes.

When group records were analyzed, the accuracy of GEBV depended on the relationships between group members, i.e. it increased when group members were more closely related, as previously reported by Olson et al. [3], Peeters et al. [1] and Su et al. [2]. Allocation of animals based on sires resulted in a higher accuracy of EBV than that based on maternal grand sires [3]. The prediction of EBV and variance components was more accurate with group records of animals from the same family than from two different families [1, 2]. A possible reason could be that a larger proportion of the phenotypic variance at the group level is explained by the additive genetic (co)variance when increasing the level of relationships between individuals within a group [2].

In addition to the increase in accuracy of GEBV by using a genomic relationship matrix in a GBLUP model, the use of genomic information resulted in additional accuracy of GEBV through optimized grouping. With the grouping methods based on genomic information proposed here, we obtained higher relationship coefficients between individuals within groups than random grouping based on pedigree, which led to a higher accuracy of GEBV when group records were used. Compared to random grouping of full-sibs, accuracy improved by 1.2 to 1.7% when genomic information was used. However, while higher accuracies are preferred, the grouping methods based on genomic information require individual genotyping before transfer of the animals to phenotype testing facilities. The small improvement in accuracy of GEBV may not offset the genotyping cost in a situation where full-sib groups can be constituted without having genomic information. For situations where only paternal half-sib groups can be produced and full-sibs cannot be identified, accuracy would improve by 11.1 to 11.7%. This relatively large increase in accuracy is mainly due to the possibility to pool full-sibs into a group with genomic information. Our approach could be useful when the objective is to obtain records on feed efficiency in a commercial testing environment or on egg production in village household chickens (e.g. a program targeting genetic gains in African Chicken [25]). Another application is for genomic selection in fish breeding programs for which mating and reproduction are natural and sib information is absent [26]. When genotyping information is available prior to group testing, grouping based on genomic information could give additional “rewards” in the form of accuracy to genomic selection in such breeding programs.

Coancestry coefficients were also lower with grouping methods based on genomic information compared to random grouping based on pedigree. In our study, the coancestry coefficients were defined as realized genomic relationships between the top 20 males and 200 females ranked by GEBV. Therefore, the coancestry coefficients are indications of future inbreeding when GEBV estimated from group records are used for selection. The use of more closely related animals to form groups can have two opposite consequences for the coancestry coefficients. One consequence is an increase in coancestry coefficients: since more closely related animals in the same group have the same phenotypic group records, GEBV between those animals are more similar, thus increasing co-selection. The other consequence is a reduction in coancestry coefficients because the use of more closely related animals to form groups increases the accuracy of GEBV prediction from group records, thus reducing co-selection. The latter benefit is obtained only with the GBLUP model because a reduction in co-selection due to increasing accuracy of prediction does not occur with pedigree-based BLUP. The EBV predicted from group records with pedigree-based BLUP are identical for full-sibs in the same group. With the prediction of GBLUP model from group records, full-sibs in the same group can have different GEBV, thus selected animals by top GEBV rank can come from different groups and different families. The effect of increasing accuracy of prediction was more pronounced when the more closely related animals were used to form groups. Thus, compared to random grouping of full or half-sibs based on pedigree, a decrease in coancestry coefficients of selected candidates was observed with grouping methods based on genomic information.

Of the two grouping methods based on genotyping information proposed here, supervised clustering based on genomic relationships had a higher accuracy, was less computationally demanding, and is more easily applied in practice than unsupervised clustering based on genotyping. Unsupervised clustering analysis with the STRUCTURE program uses genotyping data to infer population structure and assign individuals to clusters, each of these being characterized by a set of allele frequencies at each locus [8]. Criteria for inferring population structure and assigning individuals are similarity or homogeneity of alleles between individuals in clusters and Hardy–Weinberg equilibrium of alleles in clusters. With such an inferred population structure, half-sibs from each sire were assigned to one group when unsupervised clustering analysis of STRUCTURE program was applied to all offspring. Full-sibs from each family would be assigned to one group if the unsupervised clustering analysis was applied to paternal half-sibs from each sire. Therefore, unsupervised clustering analysis of STRUCTURE was applied to each full-sib family. Then, membership coefficients of individuals that belong to clusters had to be used to obtain equally sized groups. Because of this re-arrangement of animals between groups, the advantage of unsupervised clustering to pool animals with genomic similarity into groups was reduced. Unsupervised clustering based on genotypes is not ideal for assigning animals to groups because testing facilities often have a fixed capacity for group sizes and number of groups. In addition, the unsupervised clustering analysis of STRUCTURE for each full-sib family is several hundred times more computation-expensive than the grouping method of supervised clustering based on genomic relationships. In contrast, our proposed grouping method of supervised clustering assigns individuals to groups based on a genomic relationship matrix that was calculated from genotyping data. This grouping method uses a relatively simple evolutionary algorithm to cluster animals into predefined numbers of groups and desired group sizes.

Supervised clustering based on genomic relationships was carried out for each full-sib family because the probability of allocating half-sibs into the same group was very unlikely. The overlap of the distribution of full-sib and half-sib relationships is very small (Fig. 1). When supervised clustering was applied to form groups from the whole population at one time, the members within a group were always from the same full-sib family. Grouping from the whole population was time-consuming, thus only a few replicates were tested (results not shown). However, it is useful to know that when family relationships are not available to apply grouping within full-sib families, the same benefits of grouping based on genomic relationships can be obtained with additional computational effort. The same principles for animal grouping based on genomic relationships can be also applied to paternal half-sibs, half-sibs and all testing candidates when the number of full-sibs per family is smaller than the intended group sizes. Compared to grouping based on pedigree information, there is only one situation for which grouping based on genomic relationships does not lead to an increase in genomic relationships between animals within groups, i.e. when the numbers of full-sibs per family are equal to group sizes. In such a situation, grouping based on pedigree and grouping based on genomic relationships will give the same result.

The benefits of supervised clustering based on genomic information were further examined by increasing the number of surplus offspring. This approach can be useful in the situation that animals are used in different experiments, where some are done with groups, and others with individuals. This is a cost effective phenotyping strategy, and in some situations, animals raised in groups are preferred to better account for G × E interactions due to animal housing. Compared to random grouping based on pedigree information, a surplus number of offspring available for supervised clustering based on genomic information increased the genomic relationships between group members furthermore, and improved accuracy of GEBV estimated from group records. The use of supervised clustering based on genomic relationships improved the accuracy of GEBV by up to 4.5% compared to the use of random grouping of full-sibs or by 14.7% compared to the use of random grouping of paternal half-sibs.

Another factor that affected accuracy of GEBV and the genomic relationships between group members was genome structure. Genomic relationships between group members that are formed by supervised clustering depended on the genome structure, which may be related to the size of the simulated genome. Increasing the genome size decreases the genomic relationships between group members because the standard deviation of the coefficient of genomic relationships between full-sibs and between half-sibs decreases. For example, the standard deviation of genomic relationships between full-sibs is approximately equal to $\frac{0.5}{{\left( {2n_{l} } \right)^{0.5} }}$, where $n_{l}$ is the number of independent loci in the genome [5]. The standard deviation will approach zero as the number of loci increases. However, the standard deviation does not fall below about 0.035 because loci are usually linked rather than independent [5]. In addition, increasing the genome size decreases the accuracy of GEBV from GBLUP models as shown by Daetwyler et al. [27].

## Conclusions

We propose two grouping methods based on genomic information to improve the accuracy of prediction from group records. Variance components and GEBV from group records were estimated using GBLUP models. We found that estimates of variance components from group records were consistent with those from individual records and with their true values. Both grouping methods resulted in higher genomic relationships between group members, and prediction from records on these groups had a higher accuracy of GEBV prediction compared to records from random groups based on pedigree information. In addition, grouping based on genomic information led to lower coancestry coefficients of selected candidates than random grouping of paternal half-sibs and random grouping of full-sibs. Of the two proposed methods, supervised clustering based on genomic relationships was superior in terms of computation requirements and applicability. The benefits of supervised clustering based on genomic information were further examined by estimating the accuracy of GEBV from group records when the number of surplus offspring increased or when family sizes increased. Accuracy of GEBV and genomic relationship between group members that were formed by supervised clustering depended on the genome structure. In summary, genotyping information can be used to increase the accuracy of prediction from group records in two ways: genomic-based prediction and optimized grouping.

## Data Availability

The data that support the findings of this study are available from the corresponding author upon reasonable request.
